# Molecular Analysis of South African Ovine Herpesvirus 2 Strains Based on Selected Glycoprotein and Tegument Genes

**DOI:** 10.1371/journal.pone.0147019

**Published:** 2016-03-22

**Authors:** Fulufhelo Amanda Doboro, Stephen Njiro, Kgomotso Sibeko-Matjila, Moritz Van Vuuren

**Affiliations:** 1 Molecular Epidemiology and Diagnostics programme, Agricultural Research Council-Onderstepoort Veterinary Institute, Onderstepoort, Pretoria, South Africa; 2 Food, feed & Veterinary Public Health Programme, Agricultural Research Council-Onderstepoort Veterinary Institute, Onderstepoort, Pretoria, South Africa; 3 Faculty of Veterinary Sciences, University of Pretoria, Onderstepoort, Pretoria, South Africa; The University of Hong Kong, HONG KONG

## Abstract

Ovine herpesvirus 2 (OvHV-2), is the causative agent of sheep-associated malignant catarrhal fever (SA-MCF), a generally fatal disease of cattle and other captive wild ruminants. Information on the OvHV-2 strains circulating in South Africa (SA) and other African countries with regard to genetic structure and diversity, and pattern of distribution is not available. This study aimed to characterize the OvHV-2 strains circulating in SA using selected genes encoding glycoproteins and tegument proteins. To establish the genetic diversity of OvHV-2 strains, four genes, Ov 7, Ov 8 ex2, ORF 27 and ORF 73 were selected for analysis by PCR and DNA sequencing. Nucleotide and amino acid multiple sequence analyses revealed two genotypes for ORF 27 and ORF 73, and three genotypes for Ov 7 and Ov 8 ex2, randomly distributed throughout the regions. Ov 7 and ORF 27 nucleotide sequence analysis revealed variations that distinguished SA genotypes from those of reference OvHV-2 strains. Epitope mapping analysis showed that mutations identified from the investigated genes are not likely to affect the functions of the gene products, particularly those responsible for antibody binding activities associated with B-cell epitopes. Knowledge of the extent of genetic diversity existing among OvHV-2 strains has provided an understanding on the distribution patterns of OvHV-2 strains or genotypes across the regions of South Africa. This can facilitate the management of SA-MCF in SA, in terms of introduction of control measures or safe practices to monitor and control OvHV-2 infection. The products encoded by the Ov 7, Ov 8 ex2 and ORF 27 genes are recommended for evaluation of their coded proteins as possible antigens in the development of an OvHV-2 specific serodiagnostic assay.

## Introduction

Sheep-associated malignant catarrhal fever (SA-MCF) is an economically important disease that occurs world-wide [1]. Ovine herpesvirus 2 (OvHV-2) is responsible for causing SA-MCF in cattle, deer, bison and a wide variety of wild animals in captivity [2].

Mortalities resulting from SA-MCF have been reported globally including several African countries, namely, Egypt, Kenya and South Africa (SA) [3, 4, 5, 6–8, 9, 10]. Economic losses due to outbreaks of SA-MCF may occur over a long period unlike occasional infections which result in deaths of few animals at a time [11, 12]. By 2010, out of the number of MCF suspected cases in cattle received at the Agricultural Research Council-Onderstepoort Veterinary Institute (ARC-OVI) diagnostic laboratory in SA, 10% tested OvHV-2 positive while 90% tested OvHV-2 negative with PCR (M. Romito, ARC-OVI, pers. comm. 2009). In a study that involved 86 sheep from different regions of South Africa, the prevalence of OvHV-2 was found to be 77% [13]. Such a high prevalence (77%) in the natural reservoirs of OvHV-2 from different regions of SA highlights the importance of the disease in the country and necessitates investigation of the molecular epidemiology and genetic diversity of OvHV-2 strains circulating in SA. The epidemiology of SA-MCF in SA is not yet fully understood and more research on the molecular epidemiology of OvHV-2 to expand the knowledge in this field is necessary [13]. The paucity of research on OvHV-2 is due to the absence of a cell culture system to propagate the virus *in vitro*, thus resulting in difficulties studying the molecular epidemiology, diagnosis and pathogenesis of OvHV-2 [14, 15].

A genome sequence comparative study revealed sequence variations in OvHV-2 viruses occurring in different host species, cattle and sheep [16]. Apparently gene sequence variations do not occur exclusively from OvHV-2 isolates found in different hosts; Taus, et al., demonstrated sequence variations in the ORF 73 gene from isolates obtained in sheep [16]. Variations were also detected in Ov 3, Ov 10 and ORF 17 nucleotide sequences from isolates obtained from the nasal secretions of sheep [16]. Although there is evidence of genetic diversity in OvHV-2 isolates from other parts of the world, it is important to investigate the level of genetic diversity in the OvHV-2 population in cattle in South Africa (SA), which can have an impact on virulence, diagnosis, immunization against OvHV-2 and disease control.

Consequently, the aim of this investigation was to characterize OvHV-2 strains circulating in different regions of SA using genes encoding glycoproteins (Ov 7, Ov 8 ex2 and ORF 27) and the tegument protein (ORF 73). The specific genes of OvHV-2 were selected based on the predicted features of their encoded proteins such as membrane surfaced-proteins, transport activities and post-translational modifications associated with antibody binding as defined by Hart *et al*. (2007) [17]. The genetic diversity of these strains was established by PCR and DNA sequence analysis of selected OvHV-2 genes.

## Materials and Methods

### Sampling

Forty two bovine blood samples (Table 1) that previously tested OvHV-2 positive using the hemi-nested PCR directed at ORF 75 gene of OvHV-2 were made available for this project by the ARC-OVI diagnostic laboratory. The blood samples were received from different regions and specific locations of SA between the years 2007 and 2009 (Table 1). During the period of this study the approval of animal use was not needed.

**Table 1:** The list of bovine blood samples, obtained from different geographic areas in South Africa, used for molecular characterization of OvHV-2.

	Laboratory reference number	Locality of origin (SA province-Location)	Year Tested
**1**	MCF 2696	FS—Bloemfontein	2009
**2**	MCF 2721	FS—Frontfort	2009
**3**	MCF 2664	FS—Frontfort	2008
**4**	MCF 2668e	FS—Bloemfontein	2009
**5**	MCF 2666	NW—Lichtenburg	2008
**6**	MCF 2668h	FS—Bloemfontein	2009
**7**	MCF 2670	EC—Preston Park	2009
**8**	MCF 2191	EC—Queenstown	2007
**9**	MCF 2677e	NW—Rustenburg	2009
**10**	MCF 2132	MP—Middelburg	2007
**11**	MCF 2685	WC—Stellenbosch	2008
**12**	MCF 2658c	G—Cullinan	2008
**13**	MCF 2659	WC—Goodwood	2009
**14**	MCF 2658h	G—Cullinan	2008
**15**	MCF 2696	FS—Bloemfontein	2009
**16**	MCF 2661	WC—Stellenbosch	2008
**17**	MCF 2694	MP—Bronkhorspruit	2008
**18**	MCF 2643	WFS—Kimberly	2008
**19**	MCF 2645	MP—Middelburg	2008
**20**	MCF 3002.2	KZN—Cascades	2009
**21**	MCF 2709	KZN—Cascade	2009
**22**	MCF 2684	WC—Goodwood	2009
**23**	MCF 2624.1	WC—Frankfort	2008
**24**	MCF 2624.2	WC—Frankfort	2008
**25**	MCF 1987	FS—Clocolan	2007
**26**	MCF 2277.1	NW—Brandfort	2007
**27**	MCF 2040	EC—Grahamstown	2007
**28**	MCF 2195	EC—Botterfontein	2007
**29**	MCF 2127	EC—Grahamstown	2007
**30**	MCF 2140	L—Ellisras	2007
**31**	MCF 2198	WC—Vrede	2007
**32**	MCF 3002.1	KZN—Cascades	2009
**33**	MCF 2790	KZN—Cascade	2009
**34**	MCF 2850	NC—Bloemfontein	2009
**35**	MCF 2849	NC—Witrandsfontein	2009
**36**	MCF 2812	NW—Frankkfort	2009
**37**	MCF 2975	WC—Oudtshoorn	2009
**38**	MCF 2685	WC—Stellenbosch	2009
**39**	MCF 3008	KZN—Cascades	2009
**40**	MCF 3018.2	G—Pretoria	2009
**41**	MCF 3023.2	G—Elsenburg	2009
**42**	MCF 3033.1	FS—Sasolburg	2009

FS-Free State; NW- North-west; WC-Western Cape; MP-Mpumalanga; G-Gauteng; EC-Eastern Cape; L-Limpopo; KZN-Kwazulu Natal; WFS-Western Free State.

### DNA extraction

Viral DNA from the blood samples was extracted using the DNeasy Blood and Tissue kit as outlined by the manufacturer (Qiagen). An OvHV-2 negative blood sample (MCF 2222) routinely used by the ARC-OVI diagnostic laboratory was included as a negative control. No positive control was used since all the samples had previously tested positive with the hemi nested PCR targeting the ORF 75 gene of the OvHV-2.

### Primer design

Oligonucleotide primers were designed using Primer 3 [18], for the specific amplification of Ov 7, Ov 8 ex2, ORF 27, and ORF 73 genes of the OvHV-2 by PCR. The primer sequences were based on the conserved regions of the four genes as shown in Table 2. Using gene-specific primers, the estimated sizes of the PCR products for Ov 7, Ov 8 ex2, ORF 27 and ORF 73 genes were 495 bp, 253 bp, 999 bp and 1490/1649 bp (Table 2), respectively.

**Table 2:** Oligonucleotide primer sequences used for specific amplification of Ov 8 ex2, Ov 7, ORF 27 and ORF 73 genes, and indicating positions of each gene on the OvHV-2 reference genome sequences.

Gene	Sequence	Estimated PCR product size (bp)	Gene position:accession number
**Ov 7**	Forward: 5’- CAC TAT GCC CAA CTG TAT ATT GC -3’	495	80812–80834:NC007646.1 (UK)
			80812–80834:AY839756.1 (UK)
			80654–80676:DQ198083.1 (US)
	Reverse: 5’- CAT AAG CTA GGT GCT TGC—3’	495	81307–81290:NC007646.1 (UK)
			81307–81290:AY839756.1 (UK)
			81149–81132:DQ198083.1 (US)
**Ov8 ex2**	Forward: 5’- GCT AGC ACA AGG CTG GCG AGT CTA AAC -3’	253	83653–83678:NC007646.1 (UK)
			83653–83678:AY839756.1 (UK)
			83495–83521:DQ198083.1 (US)
	Reverse: 5’- TTA CTC GGT TAA ACA CAG GAC-3’	253	83906–83886:NC007646.1 (UK)
			83906–83886:AY839756.1 (UK)
			83748–83728:DQ198083.1 (US)
**ORF 27**	Forward: 5’- GTA TGG TGG GCA TAC AGA GAC TAA TC -3’	999	53995–54020:NC007646.1 (UK)
			53995–54020:AY839756.1 (UK)
			53835–53960:DQ198083.1 (US)
	Reverse: 5’- GCA CTA CAC ACA GCC AGG TTT TTC -3’	999	54994–54971:NC007646.1 (UK)
			54994–54971:AY839756.1 (UK)
			54834–54801:DQ198083.1 (US)
**ORF 73**	Forward: 5’- GTA TCC TAT TGT TGG TTA AAA GGT AAA GAT -3’	1490/1649	119041–119070:NC007646.1 (UK)
			119041–119070:AY839756.1 (UK)
			118891–118911:DQ198083.1 (US)
	Reverse: 5’- GGT GCT TTT ACG AAG TGG -3’		120531–120514:NC007646.1 (UK)
			120531–120514:AY839756.1 (UK)
			120540–120523:DQ198083.1 (US)

### PCR reactions and conditions for amplification

The amplification of Ov 7, Ov 8 ex2, ORF 27 and ORF 73 genes was carried out in a PCR reaction mixture with the final volume of 18 μℓ consisting of a HotStar HiFidelity DNA polymerase (0.5 U), MgSO_4_ (0.5 mM), dNTP (0.3 mM), forward and reverse primers (0.3 μM each), HiFidelity PCR buffer (1x), 5x Q-solution (1 x) and the DNA template of 50 ng per reaction. Except for dNTP mix, forward and reverse primers, and DNA template, all the PCR reaction components are part of the HotStar HiFidelity Polymerase Kit (Qiagen). The reaction mixtures were processed in a thermocycler following specific amplification program for each gene, as shown in Table 3.

**Table 3:** Specific thermocycling conditions for the amplification of target genes.

Gene target	Denaturing temperature, time	Annealing temperature, time	Extension temperature, time
**Ov 7**	94°C, 1 min	55°C, 1 min	72°C, 1 min
**Ov 8 ex2**	94°C, 30 s	53°C, 30 s	72°C, 30 s
**ORF 27**	94°C, 1 min	50°C, 1 min	72°C, 1 min
**ORF 73**	94°C, 2. Min	50°C, 2 min	72°C, 2.5 min

### Gel electrophoresis

Aliquots of amplicons (10 μℓ) of each of the selected genes were analyzed by gel electrophoresis using 1.5% agarose gels stained with SYBR green I nucleic acid gel stain (Roche diagnostics). Expected DNA fragments of each gene sample were excised from the agarose gel with a clean, sharp scalpel, and purified using QIAquick gel extraction kit (Qiagen) according to the manufacturer’s instruction.

### Sequencing

The purified PCR product (10–20 ng) of each gene was sent to the ARC-OVI Molecular Biology division for sequencing. Sequencing reactions were prepared using the BigDye Terminator v3.1 sequencing kit (Applied Biosystems, USA). Multiple sequence alignments were performed using the BioEdit sequence alignment [19]. Multiple sequence alignments were performed from the start to the last codon before the stop codon of the genes, for example, from region 80901 to 81263 (Accession no: NC007646.1), 53997 to 54875 (Accession no: NC007646.1) and 118890 to 120578 (Accession no: DQ198083.1) for Ov 7, ORF 27 and ORF 73, respectively. Multiple sequence alignments for the Ov 8 ex2 gene were analyzed from region 83703 to 83903 (Accession no: NC007646.1). The nucleotide sequences were edited using the BioEdit sequence alignment editor; where an ambiguity character such as N was assigned, it was replaced with a specific nucleotide base following analysis of chromatograms. The specificity of the nucleotide and protein sequences was confirmed using the Basic Local Alignment Search Tools for nucleotides and proteins, blastn and blastp, respectively. The multiple sequence alignments for each gene were produced using ClustalW. The nucleotide sequences were aligned against respective gene regions from the reference genome sequences.

The derived amino acid sequences for each gene were obtained using the ExPASy translation tool [20] and were subjected to analysis on the BepiPred 1.0 server to predict the location of B-cell epitopes [21, 22] and to determine sequence variations in the epitope regions.

### Phylogenetic analysis

The neighbour-joining and Maximum Likelihood methods were used for phylogenetic reconstruction in order to confirm the genotypes or groups of the nucleotide and amino acid sequences of the different OvHV-2 genes identified from multiple sequence alignment analyses:

#### Neighbor-Joining (NJ) method

Evolutionary analyses were conducted in MEGA 5 (Molecular Evolutionary Genetics Analysis software) [23]. The evolutionary history was inferred using the Neighbor-Joining method [24]. The bootstrap test was conducted with 1000 replicates to determine the percentages of replicate trees in which the associated taxa clustered together [25]. For nucleotide sequences, the evolutionary distances were computed using the Maximum Composite Likelihood method and are in the units of the number of base substitutions per site. For amino acid sequences, the evolutionary distances were computed using the Poisson correction method [26] and are in the units of the number of amino acid substitutions per site.

#### Maximum Likelihood (ML) method

Evolutionary analyses were also conducted in MEGA5 [27]. For the nucleotide and amino acid sequences of each gene, the evolutionary history was inferred by using the Maximum Likelihood method based on the Tamura-Nei model [28] and JTT matrix-based model [29], respectively. The percentage of trees in which the associated taxa clustered together were determined by the bootstrap test based on 1000 replicates. Initial tree(s) for the heuristic search were obtained automatically by applying Neighbor-Joining and BioNJ algorithms to a matrix of pairwise distances estimated using a JTT model, and then selecting the topology with superior log likelihood value.

## Results

### Detection of OvHV-2 genes Ov 8 ex2, Ov 7, ORF 27 and ORF 73, by PCR

**Fig 1:**
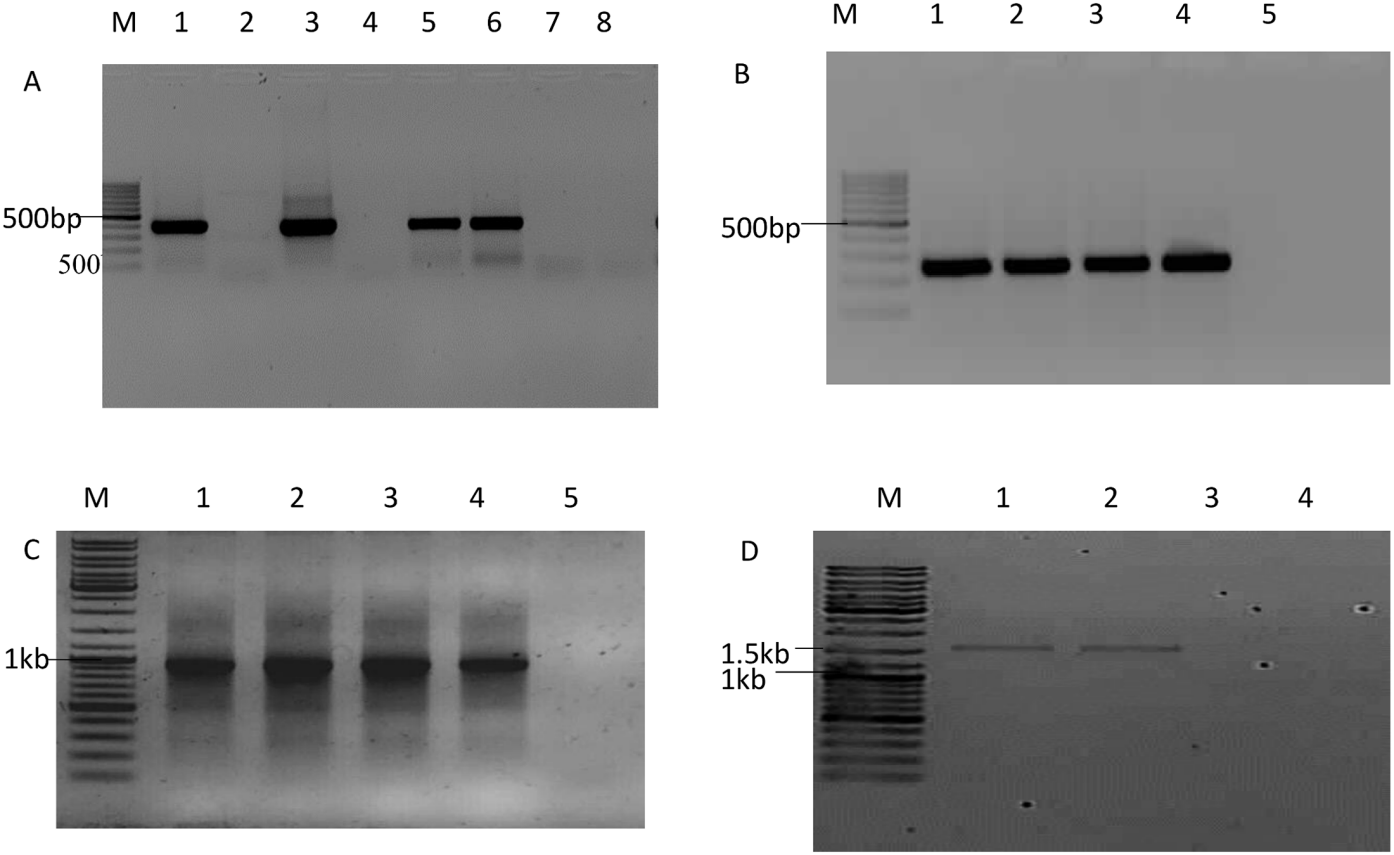
A representation of PCR products obtained from amplification of complete glycoprotein genes, Ov 7 (495 bp), Ov 8 ex2 (253 bp), ORF 27 (999 bp) and the tegument gene, ORF 73 (1649 bp) of OvHV-2.

Of the 42 blood samples investigated, Ov 7, Ov 8 ex2, ORF 27 and ORF 73 genes could be successfully amplified in 18 (43%), 38 (90%), 17 (40%) and 13 (13%) samples, respectively. The amplicon sizes obtained were as expected (Fig 1) and no amplicon was observed from the negative control samples.

### Sequence analysis

The total number of Ov 7, Ov 8 ex2, ORF 27 and ORF 73 gene amplicons that could be successfully sequenced were four (22.2%), twenty two (57.9%), five (29.4%) and three (23.1%), respectively. PCR products were amplified downstream and upstream of each of the selected gene to accommodate the whole gene, however, the multiple sequence analysis is based on sequences encompassed between the start and stop codon of each gene. Blast analysis confirmed the PCR amplicon sequences to correspond to those of Ov 7, Ov 8 ex2, ORF 27 and ORF 73 nucleotide sequences of OvHV-2. The genotypes and groups obtained with the multiple sequence analysis were confirmed with phylogenetic analysis.

### Ov 7 nucleotide and derived amino acid sequence analyses

**Fig 2:**
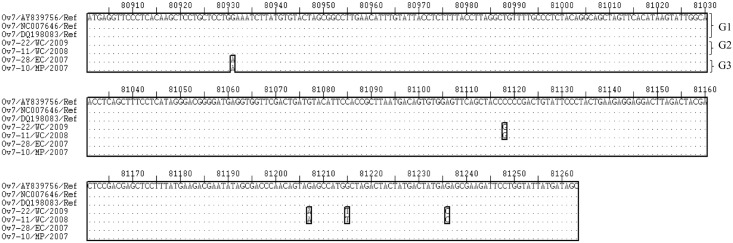
Multiple sequence alignment of nucleotide sequences of the Ov 7 gene from SA OvHV-2 strains and reference sequences derived from OvHV-2 genome sequences (Accession numbers: NC-007646.1, AY839756.1, DQ198083.1), showing three genotypes of Ov 7 gene. Ref on the alignment indicates the nucleotide sequences of the Ov 7 gene of the OvHV-2 reference genomes obtained from the GenBank. Variable regions are highlighted in rectangles (▯) and group of genotypes (G) are illustrated on the right side of the align sequences.

**Fig 3:**
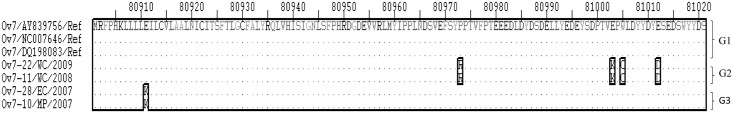
Multiple sequence alignment of amino sequences of the Ov 7 gene from SA OvHV-2 strains and reference sequences derived from OvHV-2 genome sequences (Accession number: NC-007646.1, AY839756.1, DQ198083.1) analysed with ExPASy translate tool. Ref on the alignment indicates the amino acid sequences of the Ov 7 gene of the OvHV-2 reference genomes obtained from the GenBank. Variable regions are highlighted in rectangles (▯) and groups of amino acid sequences (G) are illustrated on the right side of the align sequences.

**Fig 4:**
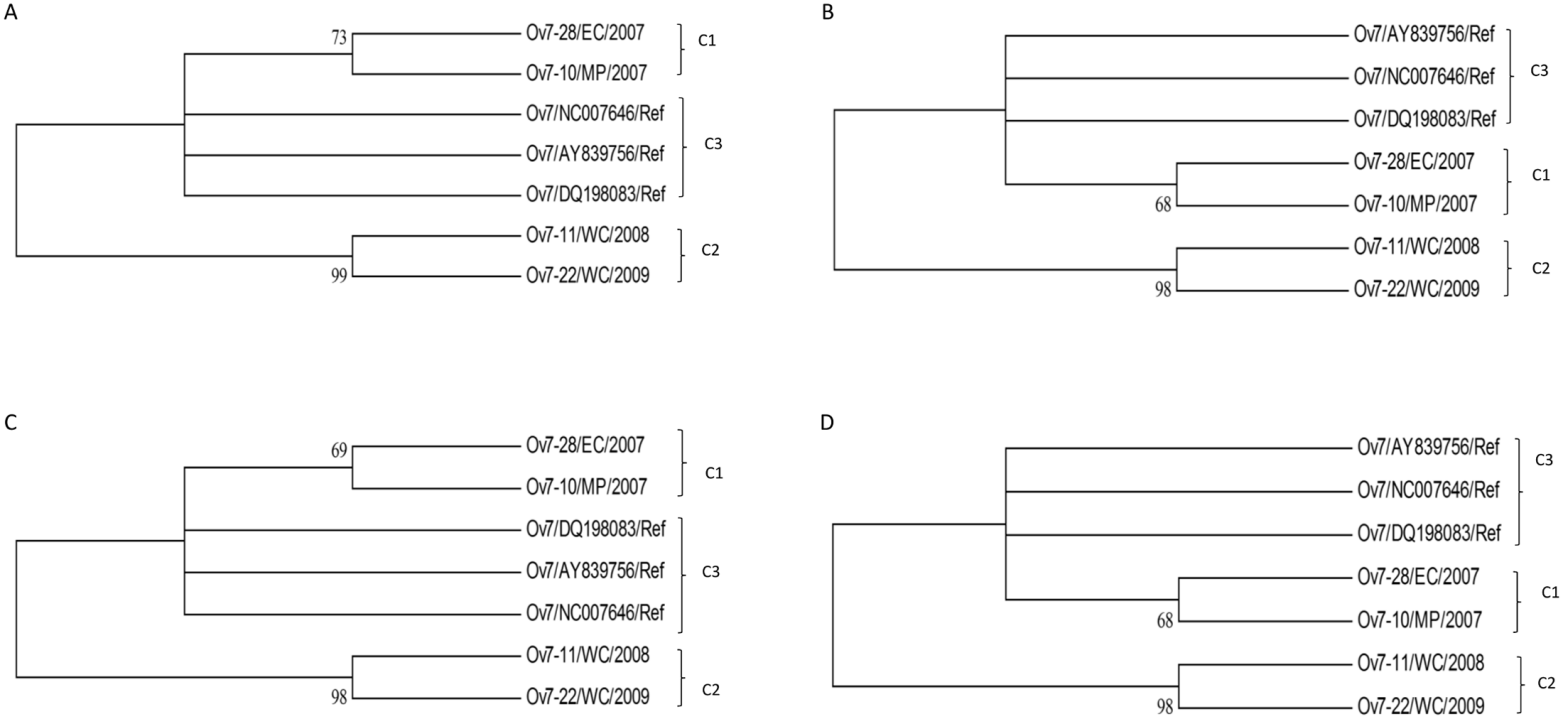
The phylogenetic trees of the Ov 7 nucleotide (A & B) and amino acid (C & D) sequences of OvHV-2 strains and their closely related reference sequences obtained from GenBank. The unrooted condensed trees were constructed using Neighbor-Joining (A & C) and Maximum Likelihood (B & D) methods, showing branching patterns of each clade. Groups 1, 2 and 3 are illustrated and abbreviated as clade 1 (C1), 2 (C2) and 3 C3), respectively, with supporting bootstrap values at each clade nodes.

The 363 bp nucleotide and 121 derived amino acid (aa) sequences of the complete Ov 7 gene were used to produce multiple sequence alignments with Ov 7 reference gene sequences (accession numbers: NC-007646.1, AY839756.1 and DQ198083.1) (Figs 2 and 3, respectively). Phylogenetic trees were constructed using 200 bp nucleotide and 67 derived amino acid sequences of the Ov 7 (Fig 4).

Nucleotide and amino acid sequence variations were observed between the Ov 7 gene sequences from SA samples and that of reference sequences (Figs 2 and 3, respectively). Comparing SA and reference genes, Ov 7 nucleotide and amino acid sequence identities ranged between 98.8–99.7% and 96.6–99.1%, respectively (S1 Table), and between SA sequences, 98.6–100% and 95.8–100%, respectively (S1 Table).

Analysis of the multiple sequence alignment of Ov 7 nucleotide sequences revealed single nucleotide sequence differences at five positions, 80931, 81118, 81207, 81215 and 81236 (Fig 2). These single nucleotide polymorphisms (SNPs) resulted in the identification of three genotypes of Ov 7 sequences that were designated as genotype 1 consisting of the three reference sequences, NC-007646.1, AY839756.1 and DQ198083.1, genotype 2 consists of SA sequences Ov7-22/WC/2009 and Ov7-11/WC/2008 and genotype 3 consists of two SA sequences, Ov7-28/EC/2007 and Ov7-10/MP/2007. Notably, Ov7-28/EC/2007 and Ov7-10/MP/2007 sequences were very similar to reference sequences with only one SNP difference.

Similar to nucleotide sequences, three major groups of Ov 7 amino acid sequences were identified from the analysis of the multiple sequence alignment between SA and reference sequences (Fig 3). These groups consisted of the same taxa as observed in the nucleotide multiple sequence alignment thus designated genotype 1, 2 and 3 with respect to the nucleotide sequence genotypes consisting of the same sequences. The single amino acid sequence variations were observed at three positions, 80973, 81003, and 81012, for group 1, and at position 80911 for group 2. Other than SNPs, no major sequence variations were observed among the nucleotide or amino acid sequences of the Ov 7 genes.

Phylogenetic analysis of Ov 7 nucleotide and derived amino acid sequences revealed three clades, with group classification similar to that identified from the multiple sequence alignment analysis (Fig 4). The different clades were well supported with good bootstrap values at each clade nodes (Fig 4).

### Ov 8 ex2 nucleotide and derived amino acid sequence analysis

**Fig 5:**
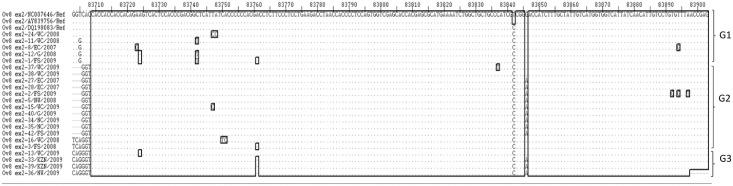
Multiple sequence alignment of nucleotide sequences of the Ov 8 ex2 gene from SA OvHV-2 strains and reference sequences derived from OvHV-2 genome sequences (Accession numbers: NC-007646.1, AY839756.1, DQ198083.1), showing three genotypes of Ov 8 ex2 gene. Ref indicates the nucleotide sequences of the Ov 8 ex2 gene of the OvHV-2 reference genomes obtained from the GenBank. Variable regions are highlighted in rectangles (▯), and groups of genotypes (G) are illustrated on the right side of the align sequences.

**Fig 6:**
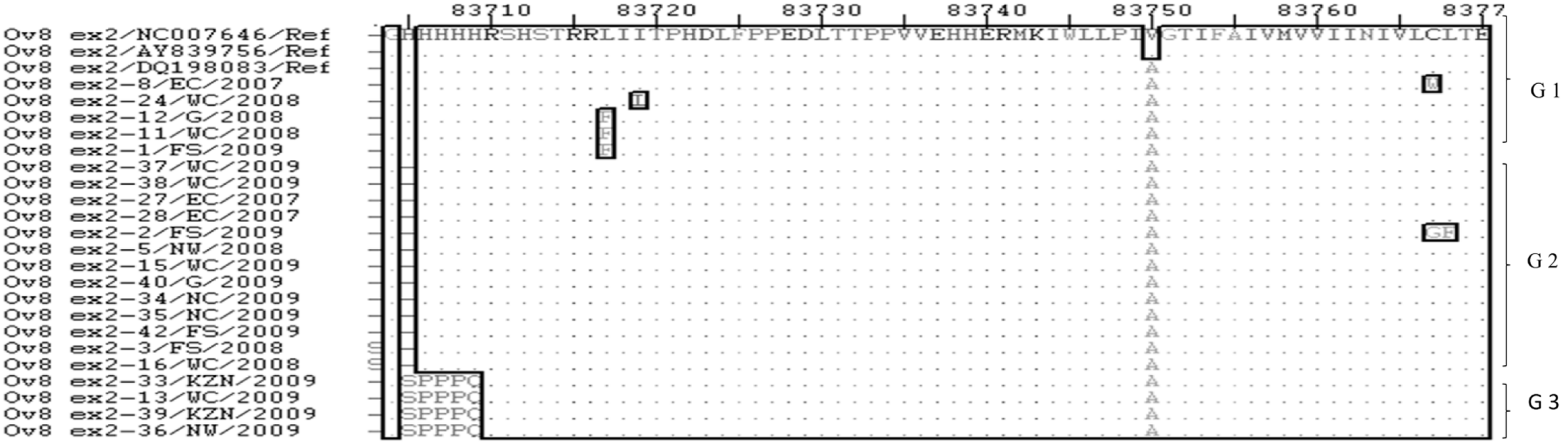
Multiple sequence alignment of amino acid sequences of the Ov 8 ex2 gene from SA OvHV-2 strains and reference sequences derived from OvHV-2 genome sequences (Accession numbers: NC-007646.1, AY839756.1, DQ198083.1) analyzed with ExPASy translate tool. Ref indicates the amino acid sequences of the Ov 8 ex2 gene of the OvHV-2 reference genomes obtained from the GenBank. Variable regions are highlighted in rectangles (▯), and groups of amino acid sequences (G) are illustrated on the right side of the align sequences.

**Fig 7:**
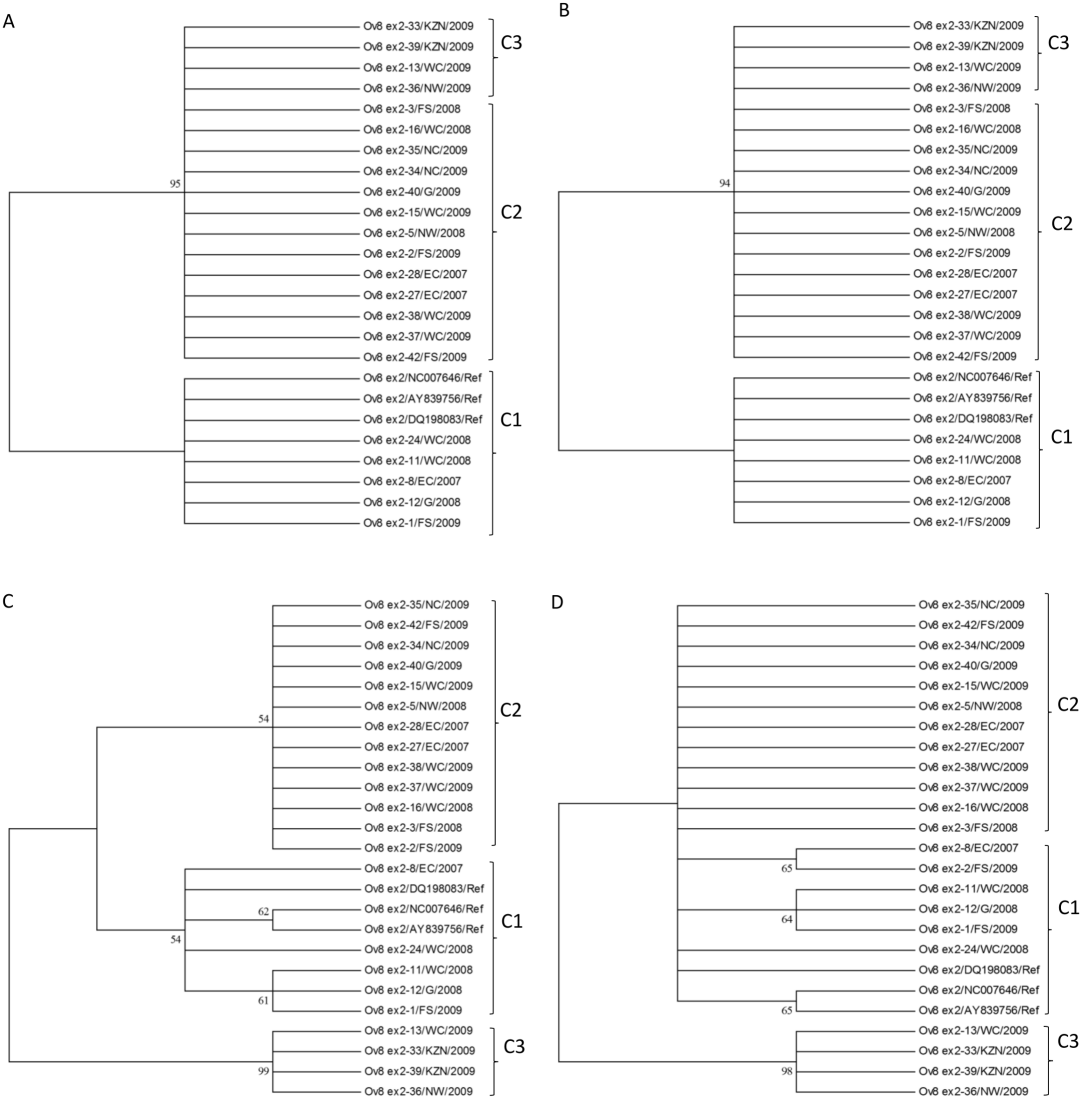
The phylogenetic trees of the Ov 8 ex2 nucleotide (A & B) and amino acid (C & D) sequences of OvHV-2 strains and their closely related reference sequences obtained from GenBank constructed using Neighbor-Joining (A & C) and Maximum Likelihood (B & D) methods. The condensed trees constructed using the Maximum Likelihood (B & D) methods indicate the branching patterns of each clade. The unrooted condensed trees were constructed using Neighbor-Joining (A & C) and Maximum Likelihood (B & D) methods, showing branching patterns of each clade. Groups 1, 2 and 3 are illustrated and abbreviated as clade 1 (C1), 2 (C2) and 3 C3), respectively, with supporting bootstrap values at each clade nodes.

Multiple sequence alignments with Ov 8 ex2 gene reference sequences were produced from the 200 bp nucleotide and 67 derived amino acid sequences (Figs 5 and 6, respectively) obtained from Ov 8 ex2 gene amplicons. Phylogenetic trees were also obtained using similar sizes of 200 bp nucleotide and 67 derived amino acid sequences of the Ov 8 ex2 (Fig 7).

The nucleotide sequence identity between SA and reference Ov 8 ex2 sequences ranged between 92.5 and 99%, and among SA sequences was between 92 and 100% (S2A Table). The amino acid sequences identity ranging from 91 to 98.5%, was obtained between SA and Ov 8 ex2 gene reference sequences, and 89.5 to 100% among SA Ov 8 ex2 sequences (S2B Table).

Nucleotide sequence variations observed resulted in three groups of Ov 8 ex2 sequences (Fig 5). The three groups are characterized by nucleotide motifs ‘CGTCAC’, ‘---GGT’, ‘TCAGGT’ or ‘CAGGGT’ between positions 83703–83708 and were respectively designated Ov 8 ex2 genotypes 1, 2 and 3. Ov 8 ex2 genotype 1 sequences were shared between the references and some of the SA OvHV-2 sequences. SNPs were observed at 12 positions of the Ov 8 ex2 aligned nucleotide sequences, however only few were non-synonymous, affected the predicted protein sequence (Figs 5 and 6). These Ov 8 ex2 sequence groups identified from the nucleotide multiple sequence alignment were retained in the amino acid multiple sequence alignment (Fig 6). Major sequence variations occurred at the 5’-end within six nucleotides among the nucleotide sequences of the Ov 8 ex2 genes and same variations were retained at the N-terminal of the amino acid sequences.

Phylogenetic trees using Ov 8 ex2 nucleotide and derived amino acid sequences confirmed the three genotypes revealed in three clades with good supporting bootstrap values at each clade nodes (Fig 7).

### ORF 27 nucleotide and derived amino acid sequence analysis

**Fig 8:**
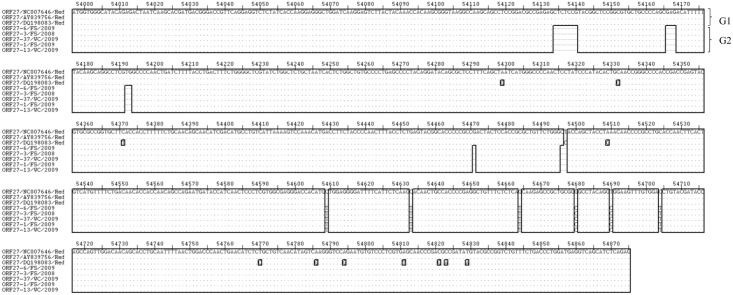
Multiple sequence alignment of nucleotide sequences of the ORF 27 gene from SA OvHV-2 strains and reference sequences derived from OvHV-2 genome sequences (Accession numbers: NC-007646.1, AY839756.1, DQ198083.1), showing two genotypes of ORF 27 gene. Ref indicates the nucleotide sequences of the ORF 27 gene of the OvHV-2 reference genomes obtained from the GenBank. Variable regions are highlighted in rectangles (▯), and groups of genotypes (G) are illustrated on the right side of the align sequences

**Fig 9:**
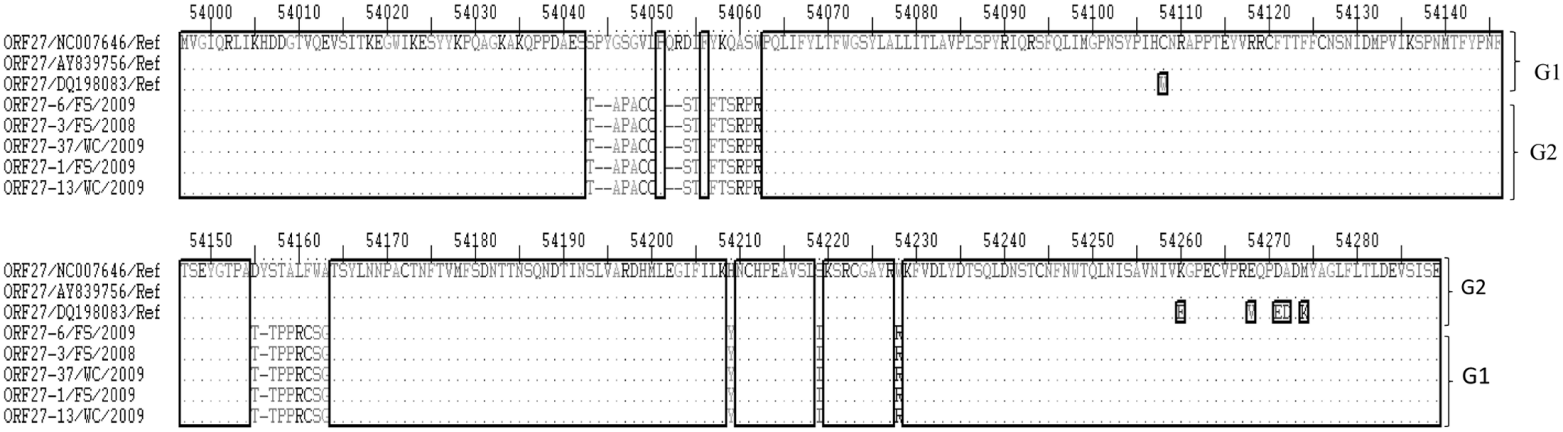
Multiple sequence alignment of amino acid sequences of the ORF 27 gene from SA OvHV-2 strains and reference sequences derived from OvHV-2 genome sequences (Accession numbers NC-007646.1, AY839756.1, DQ198083.1) analyzed with ExPASy translate tool. Ref indicates the amino acid sequences of the ORF 27 gene of the OvHV-2 reference genomes obtained from the GenBank. Variable regions are highlighted in rectangles (▯), and groups of amino acid sequences (G) are illustrated on the right side of the align sequences.

**Fig 10:**
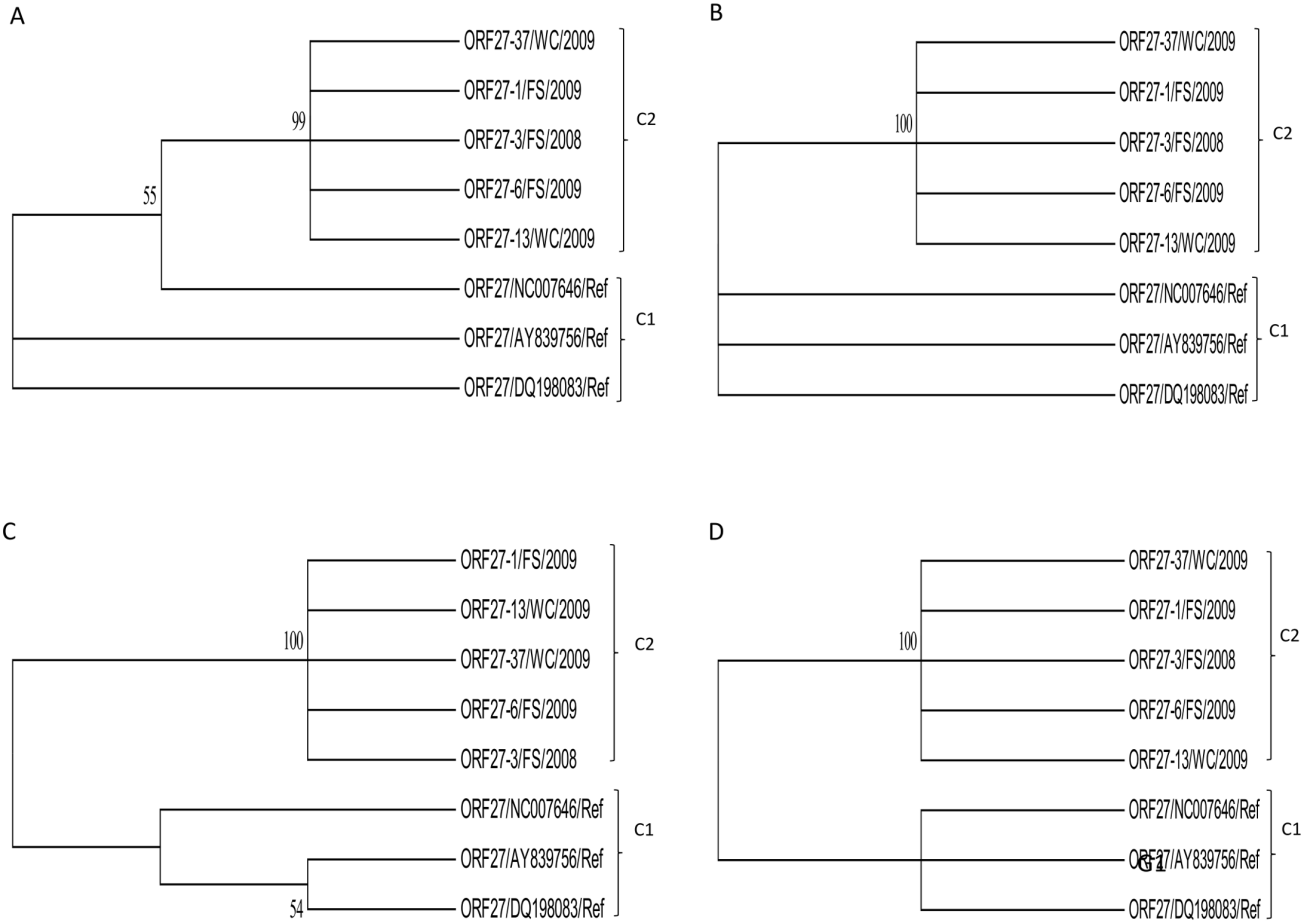
The phylogenetic trees of the ORF 27 nucleotide (A & B) and amino acid (C & D) sequences of OvHV-2 strains and their closely related reference sequences obtained from GenBank constructed using Neighbor-Joining (A & C) and Maximum Likelihood (B & D) methods. The unrooted condensed trees were constructed using Neighbor-Joining (A & C) and Maximum Likelihood (B & D) methods, showing branching patterns of each clade. Groups 1 and 2 are illustrated and abbreviated as clade 1 (C1) and 2 (C2), respectively, with supporting bootstrap values at each clade nodes.

The 878 bp nucleotide and 292 derived amino acid (aa) sequences of the ORF 27 gene were used to generate multiple sequence alignments including sequences from SA OvHV-2 strains and the reference genomes (Figs 8 and 9, respectively). Based on the 200 bp nucleotide and 67 derived amino acid sequences of the ORF 27, phylogenetic trees were obtained (Fig 10).

The nucleotide and amino acid sequence identities of the ORF 27 gene ranged from 96.3 to 97.6% and 87.7 to 89.7%, respectively (S3 Table). Notable was the 100% sequence identity obtained for SA sequences on both the nucleotide and amino acids (S3 Table).

Multiple sequence analysis of ORF 27 gene nucleotide and amino acid sequences revealed two groups of sequences designated ORF 27 genotypes 1 and 2 (Figs 8 and 9). Genotype 1 consists of reference sequences while all SA ORF 27 sequences were identified as ORF 27 genotype 2. According to the nucleotide multiple sequence analysis, the ORF 27 genotype 2 is characterized by nucleotide deletions at 5 positions of the gene sequence, including positions 54134, 54140, 54166–54168, 54192–54193, 54471, and 54496–54497. Furthermore, genotype 2 is characterized by SNPs at positions 54609, 54633, 54664 and 54704.

In the amino acid multiple sequence alignment of ORF 27 amino acid sequences, two groups similar to that observed in the nucleotide sequences were obtained. ORF 27 group 2 sequences were characterized by amino acid changes [‘T’ at position 54043, ‘ST’ at position 54054 and 54055, ‘T’ at position 54155, and, ‘Y’, ‘I’, and ‘R’ at positions 54209, 54219 and 54228, respectively], deletions [‘PY’ at 54044 and 54045, ‘QR’ at 54052 and 54053, ‘Y’ at 54156] and amino acid motifs[‘APACC’ from position 54046 to 54050, ‘FTSRPR’ from position 54057 to 54062 and ‘TPPRCSG’ from position 54157 to 54163] (Fig 9). Variation occurred often and randomly among the nucleotide sequences of the ORF 27 genes and rarely at the C-terminal among amino acid sequences of the glycoprotein encoded by ORF 27.

Phylogenetic analysis using ORF 27 nucleotide and derived amino acid sequences revealed two clades, namely clade 1 and 2, with good supporting bootstrap value (s) at each clade nodes (Fig 10).

### ORF 73 nucleotide and derived amino acid sequences analysis

**Fig 11:**
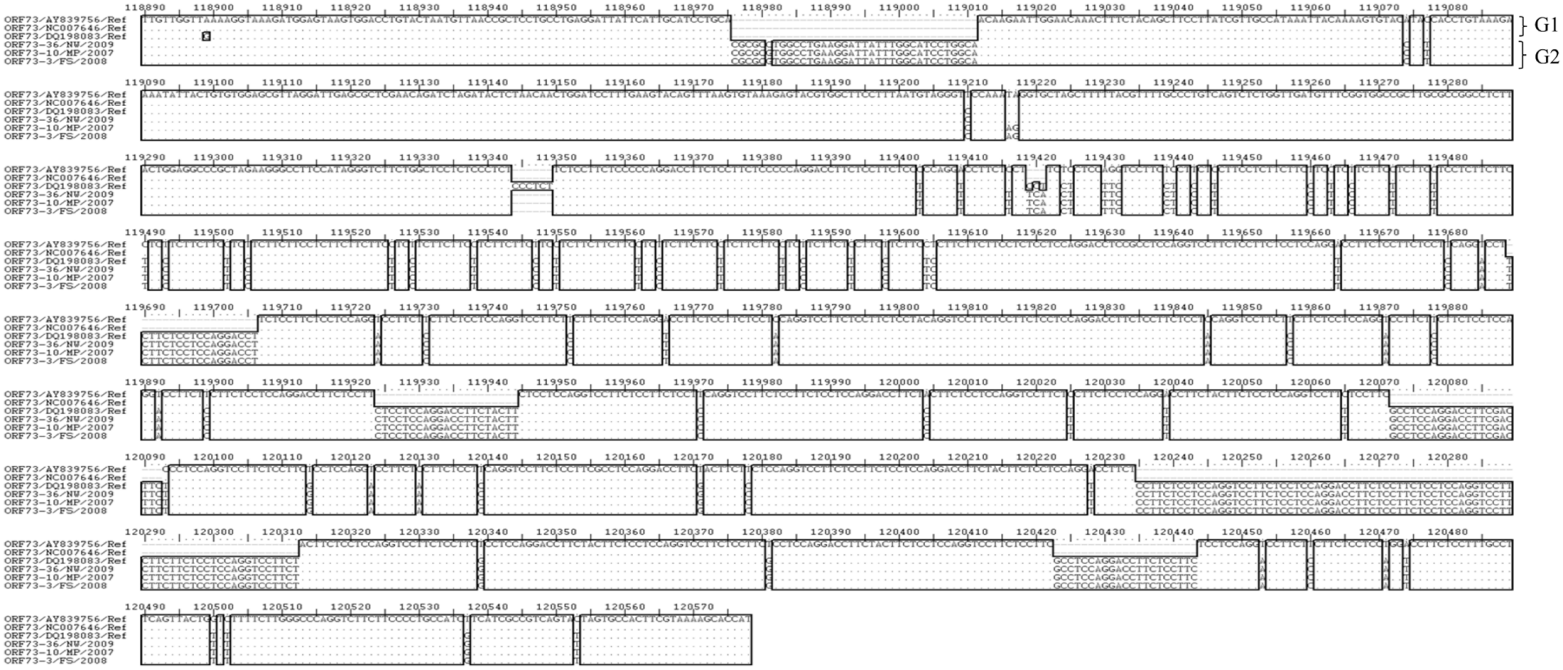
Multiple sequence alignment of nucleotide sequences of the ORF 73 gene from SA OvHV-2 strains and reference sequences derived from OvHV-2 genome sequences (Accession numbers NC-007646.1, AY839756.1, DQ198083.1). Ref indicates the nucleotide sequences of the ORF 73 gene of the OvHV-2 reference genomes obtained from the GenBank. Variable regions are highlighted in rectangles (▯), and groups of genotypes (G) are illustrated on the right side of the align sequences.

**Fig 12:**
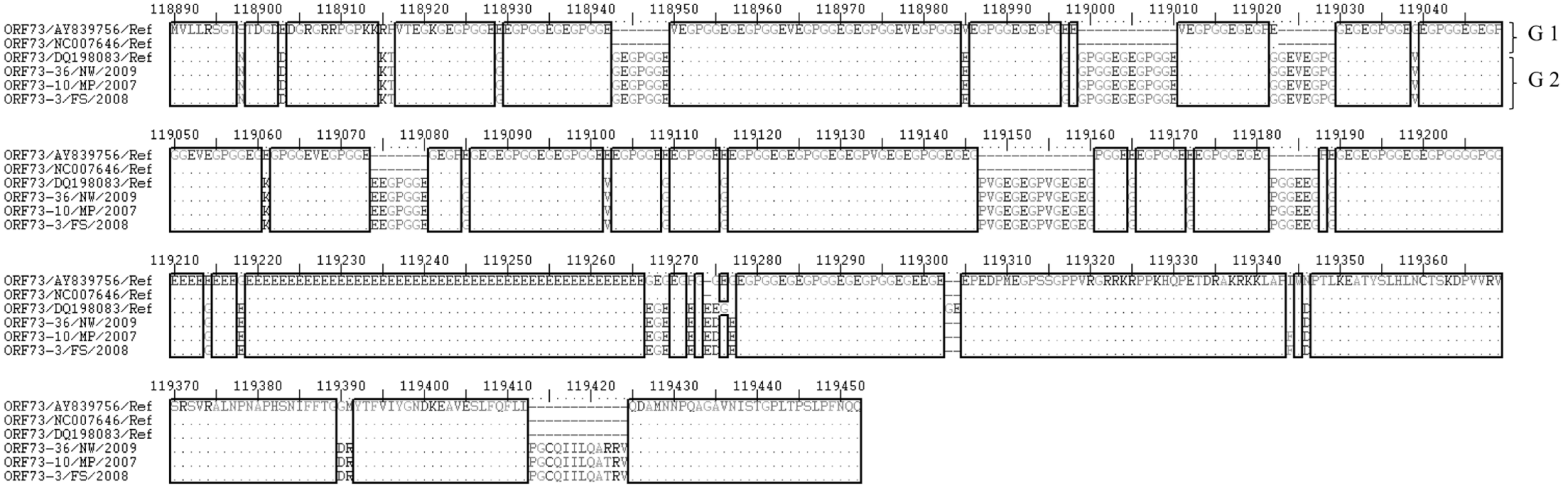
Multiple sequence alignment of amino acid sequences of the ORF 73 gene from SA OvHV-2 strains and reference sequences derived from OvHV-2 genome sequences (Accession numbers: NC-007646.1, AY839756.1, DQ198083.1) analysed with ExPASy translate tool. Ref indicates the amino acid sequences of the ORF 73 gene of the OvHV-2 reference genomes obtained from the GenBank. Variable regions are highlighted in rectangles (▯), and groups of amino acid sequences (G) are illustrated on the right side of the align sequences

**Fig 13:**
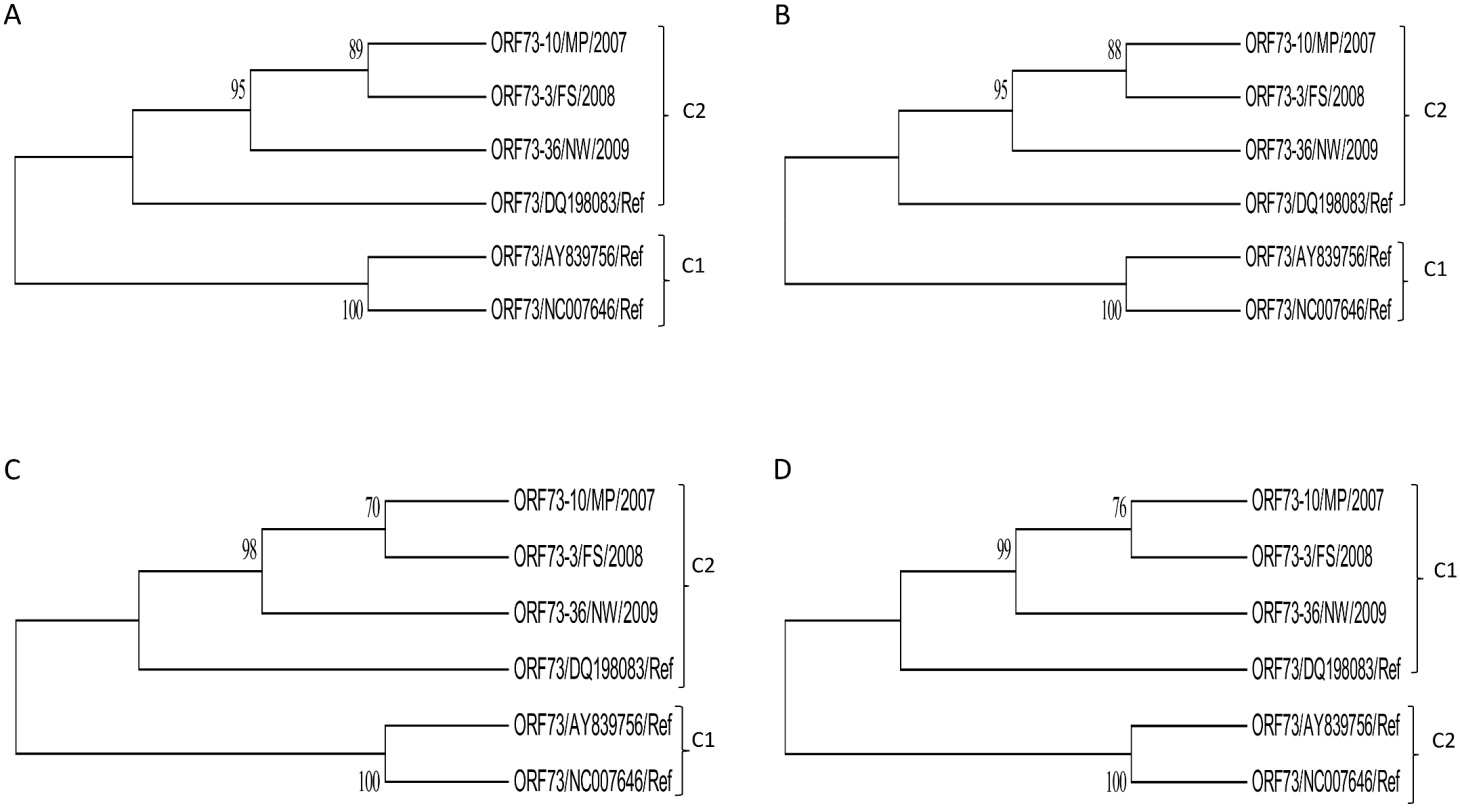
The phylogenetic trees of the O RF 73 nucleotide (A & B) and amino acid (C & D) sequences of OvHV-2 strains and published closely related reference sequences obtained from GenBank constructed using Neighbor-Joining (A & C) and Maximum Likelihood (B & D) methods. The unrooted condensed trees were constructed using Neighbor-Joining (A & C) and Maximum Likelihood (B & D) methods, showing branching patterns of each clade. Groups 1 and 2 are illustrated and abbreviated as clade 1 (C1) and 2 (C2), respectively, with supporting bootstrap values at each clade nodes.

The sizes of the aligned nucleotide and derived amino acid sequences of ORF 73 gene were 1688 bp (Fig 11) and 562 bp (Fig 12), respectively. The actual size of the ORF 73 for SA strains were larger than the expected estimated size because of the large random insertion mutations observed in the SA ORF 73 sequences (Fig 11). The nucleotide and amino acid sequence identity of the ORF 73 gene between SA and references sequences ranged between 83.6 to 97.1% and 83 to 96.6%, respectively (S4 Table). Among SA sequences the sequence identity ranged between 99.8 to 100% for nucleotides and 99.6 to 100% for amino acids (S4 Table). Phylogenetic trees constructed using 200 bp nucleotide and 67 derived amino acid sequences of the ORF 73 were obtained (Fig 13, respectively).

Sequence variations were observed on the ORF 73 nucleotide and amino acid sequences between the SA and OvHV-2 reference sequences. Two groups of sequences were identified from ORF 73 sequences analyzed, and were designated genotypes 1 and 2. Genotype 1 consists of reference sequences, AY839756.1 and NC007646.1 and genotype 2 is composed of all SA ORF 73 sequences and reference sequence DQ198083.1. Genotypes 1 and 2 are distinguished from each other by nucleotide deletions from position 118976 to 119011 and insertions at position 119074 and 119077 (Fig 11). Genotype 2 sequences were all identical, except DQ198083.1 sequence which was marked by several nucleotides and amino acid sequence differences of which some were characteristic of genotype 1 sequences. The same number of groups corresponding to the nucleotide genotypes were retained in amino acid multiple sequence analysis (Fig 12). From the amino acid analysis, group 2 sequences obtained from SA strains are characterized by motifs ‘PGCQIILQARRV’ from sample ORF73-36/NW/2009 and ‘PGCQIILQATRV’ from ORF73-10/MP/2007 and ORF73-3/FS/2009, at position 119413 to 119424 and amino acid ‘DR’ at position 119390 to 119391. Furthermore, SA and two reference sequences (AY839756.1 and NC007646.1) are distinguished by amino acids ‘E’, ‘I’, ‘E’ ‘D’ and ‘R’, respectively at positions 118898 and 119346.

Phylogenetic analysis using both ORF 73 nucleotide and derived amino acid sequences revealed two clades (Fig 13) with supporting bootstrap values above 70 at each clade nodes.

### Distribution of genotypes in different regions at specific periods

Random distribution of genotypes was observed in the following manner; Ov 7 gene sequences: Only genotype 2 was identified in samples from SA, specifically from WC, EC and MP (Table 4), at all time periods of sample collection. Due to absence of PCR products from samples from FS, NW, G, and KZN there was no data produced for these regions. Ov 8 ex2 gene sequences: All Ov 8 ex2 genotypes were found in SA samples with genotypes 1 and 2 dominating (Table 4).

**Table 4:** Summary of genotypes of different genes investigated for characterization of South African OvHV-2 strains.

Province	Genotypes detected			
	Ov 7	Ov 8 ex2	ORF 27	ORF 73
**WC**	2	1, 2, 3	2	-
**EC**	2, 3	1, 2	-	-
**FS**	-	1, 2	2	2
**NW**	-	2, 3	-	2
**MP**	2, 3	-	-	2
**G**	-	1, 2	-	-
**KZN**	-	3	-	-

Numbers 1, 2 and 3 represent genotypes 1, 2 and 3 of the specific gene.— **= not analysed.**

Genotype 3 was only detected from samples collected in 2009 (Table 5). ORF 27 gene sequences: Only samples from two provinces (WC and FS) were investigated and from both provinces genotype 2 was identified from all time periods of sample collection (2008 and 2009) (Table 5). Samples from EC, NW, MP, G, and KZN provinces did not have amplicons that could be analysed. ORF 73 gene sequences: Samples from three provinces were analysed for ORF 73 gene (FS, MP and NW) from which genotype 2 was found, at all periods of sample collection (Table 5). Samples from EC, G, KZN and WC provinces did not have amplicons that could be analysed.

**Table 5:** Summary of genotype distribution among South African OvHV-2 strains collected at different time periods.

Year of sample collection	Genotypes detected			
	Ov 7	Ov 8 ex2	ORF 27	ORF 73
**2007**	3	1, 2	-	2
**2008**	2	1, 2	2	2
**2009**	2	1, 2, 3	-	2

Numbers 1, 2 and 3 represent genotypes 1, 2 and 3 of the specific gene.— = not analysed.

### B cell epitopes mapping of Ov 7, Ov 8 ex2 and ORF 27 genes

B cell epitope prediction analysis revealed five sites of B cell epitopes in Ov 7 and four in ORF 27 amino acid sequences while one site was observed from the Ov 8 ex2 sequences (Table 6). In all the genes investigated, these sites seem to be conserved as they occur where there are no sequence variations.

**Table 6:** Epitope mapping using derived amino acid sequences of Ov 7, Ov 8 ex2 and ORF 27 genes.

Gene	Epitope region on the multiple aligned derived amino acid sequences				
	E1	E2	E3	E4	E5
**Ov 7**	80947–80953	80964–80970	80975–80987	80995–81002	81013–81020
**Ov 8 ex2**	83721–83733	-	-	-	-
**ORF 27**	54012–54017	54030–54042	54109–54116	54149–54154	

E = Epitope; N/A = Not analysed;— = No epitope

## Discussions

In spite of the importance of SA-MCF, no research has been conducted on the molecular characterization of OvHV-2 strains in cattle in South Africa, or in other African countries. This scarcity of research in OvHV-2 in South Africa could be due to the fact that the virus has been considered dominant in non-African countries [30] whilst a closely related virus, namely, AIHV-1 was confirmed dominant in African countries due to the presence of wildebeest, which is a carrier of AIHV-1 in Africa [30].

In SA, farmers who breed sheep or wildebeest often have to deal with legal actions taken against them when cases of SA-MCF and WD-MCF occur in cattle in neighbouring farms [31]. The importance of conducting research on OvHV-2 in Africa cannot be over-emphasised and it is evident that OvHV-2 is also being recognised in Africa. Furthermore, there are no OvHV-2 isolates available for research due to challenges with the virus isolation [14], conveniently the availability of the complete genome sequence of the OvHV-2 [17] has made it possible to conduct molecular studies of this virus.

Characterizing viruses based on geographical origin is important in order to gain a better understanding of the existing genetic variation for development of specific diagnostic tools and effective vaccines. Consequently an attempt was made to characterize OvHV-2 strains circulating in the cattle population in SA, based on four OvHV-2 genes, namely, Ov 7, Ov 8 ex2, ORF 27 and ORF 73.

### Amplification of selected OvHV-2 genes

Detection of OvHV-2 DNA using PCR has since become a preferred method of diagnosis over histopathology test and is globally recognized [32, 33]. Most of the diagnostic laboratories have adopted PCR assays developed from previous research studies targeting the specific genes of OvHV-2 [32, 34, 35, 36]. Nested and real-time PCR have been adopted to screen OvHV-2 in clinical and subclinical cases [34, 37]. Hence, PCR was employed to amplify selected genes of OvHV-2, namely Ov 7, Ov 8 ex2, ORF 73 and ORF 27, for characterization of OvHV-2 strains occurring in cattle in SA.

Availability of OvHV-2 genomic sequences obtained from the GenBank assisted in the development of gene-specific primers (16, 17). The primers successfully amplified the targeted regions in each gene, but the amplification was not successful in all the samples analyzed. The discrepancies in sample amplification by the different assays could have been due to low primer sensitivity, specificity, suboptimal PCR conditions or poor DNA sample quality. Several reasons affect the reproducibility of PCR results ranging from the quality of the blood sample, DNA extraction methods, amount and quality of nucleic acid template and PCR reaction components to thermocycling conditions [38]. Although it was not in the interests of the objectives of the study to optimize the different PCR assays used, the PCR sensitivity could have been improved using fresh positive OvHV-2 blood samples for DNA extraction, a different DNA extraction method for better DNA quantities, re-design amplification primers, test different temperatures for optimal primer annealing and/or use a more sensitive Taq polymerase. However, collection of fresh OvHV-2 positive blood samples during the period of this work was not practical, because the study was focused on clinical samples that had previously tested positive for the OvHV-2 virus using an existing discriminative PCR method. Field samples would still require screening of OvHV-2 positive samples from an unknown population of samples using an existing PCR method because the designed primers and protocol could not be used for screening purposes as they were still under optimization. Nonetheless, PCR products of the four genes were further analyzed by DNA sequencing to characterize OvHV-2.

### Sequences analysis

Nucleotide and amino acid multiple sequence alignment analyses revealed two genotypes identified from ORF 27 and ORF 73 nucleotide sequences, and three genotypes from Ov 7 and Ov 8 ex2 nucleotide sequences. The genotypes and groups observed with phylogenetic analysis using Ov 7, Ov 8.2, ORF 27 and ORF 73 nucleotide and derived amino acid sequences, respectively, were in agreement with the number of genotypes and groups obtained with multiple sequence analysis.

Sequence diversity has not been previously demonstrated in Ov 7, Ov 8 ex2 and ORF 27 genes; however, genetic diversity has previously been demonstrated in Ov 3, Ov 10, ORF 73 and ORF 17 genes of OvHV-2 [16]. ORF 73 gene sequence diversity has been demonstrated between the OvHV-2 strains from the nasal secretions of sheep [16] and the lymphoblastoid cell line of the BJ1035 cow [17]. Furthermore, variation in the sequences of the ORF 73 gene has been used for subtyping Kaposi’s sarcoma associated herpesvirus (KSHV) and to study the variability among the KSHVs [39, 40].

Genetic mutations in the form of SNPs, nucleotide insertions and deletions were responsible for the variation observed from each gene. Nucleotide insertions and deletions are types of mutations that have been observed in other viruses including equine gammaherpesvirus (γ-EHV) [41, 42] and the OvHV-2 closely related AIHV-1. In the latter, mutations caused by deletion or re-assortments of the genome were due to continuous passage in cell culture which resulted in pathogenic wild strains becoming non-pathogenic or attenuated [43].

Point mutations are the most common types of mutations observed in nucleotide sequences of the genes investigated, including synonymous and non-synonymous mutations, with the latter being the most common. Since synonymous mutations do not affect the protein, this type of mutation has no real role in the evolution of species [44]. On the contrary, non-synonymous mutations (Deletion, insertion and SNP) affect the amino acids that are coded for and change the resulting protein that is expressed (44). The severity of this kind of mutation depends on where in the amino acid sequence the mutation occurs. If it occurs at the amino terminal, the entire protein is changed; this could affect the function of this protein [45]. However, non-synonymous mutations can be beneficial in that they increase the diversity in the gene pool thus encouraging for natural selection and drive evolution on a micro-evolutionary level [46]. None of the non-synonymous mutations in the genes investigated occurred on the N-terminal of the protein. Furthermore, the mutations did not occur in the B cell epitope region of the proteins thus the antibody-binding activity of these proteins is not affected. Therefore, implications due to variation in the sequence observed on each gene suggested that ORF 27 and ORF 73 genes can be used to differentiate OvHV-2 strains based on the observed unique patterns of sequence for each genotype, and can be used to study the OvHV-2 strains diversity. Nonsense mutation, another type of mutation which results in a codon that translated into a stop signal, was not observed from the genes investigated.

The importance of nucleotide and amino acid sequence variations in viruses is not only limited to understanding genetic diversity, they also provide insight into virulence and pathogenesis of a particular virus, and information for development of diagnostic tools and effective antiviral drugs and vaccines. Currently the pathogenesis of OvHV-2 is unknown [47]. Due to lack of sufficient clinical history of the animals used, the genotypes that were identified could not be directly linked to the virulence of OvHV-2. However, two different blood samples obtained from two naturally infected bovines that died from OvHV-2 infection were confirmed by PCR test specific for SA-MCF at the ARC-OVI laboratory, and supported by the presence of clinical signs of MCF recorded on the registration form. Genotypes 1 and 2 of the Ov 8 ex2 and ORF 27 genes were identified, respectively. Although OvHV-2 does not always cause disease in cattle, deer and bison [2, 48], findings from this study have shown that OvHV-2 strains consisting of genotypes 1 and 2 of the Ov 8 ex2 and ORF 27 genes, respectively, are virulent, at least to cattle.

Genetic diversity can also be influenced by geographical location because of environmental conditions such as climate, temperature or weather [49]. Genetic variations linked to geographic origin are significant in the manifestation of the disease particularly where weather patterns vary significantly [49]. Variations in the sequences of the genome associated with different geographic origin of the viral strains have been observed in other viruses including Fibropapilloma-associated marine turtle herpesvirus (FPTHVS) equine gammaherpesvirus 2 (EHV-2) and 5 (EHV 5) strains [50, 51]. Sequence analysis of Ov 7 and ORF 27 revealed variations that distinguished between the SA and reference OvHV-2 strains, which originate from the United Kingdom (UK) and United State of America (USA). However, more samples should be tested from the UK and USA to confirm this result. The information on genotypes linked to geographic origin will be essential since it can be used to trace the origin of infection when an outbreak occurs, particularly in areas that have previously never experienced an OvHV-2 outbreak.

There are no effective control measures against MCF in SA [31, 48]. Prevention of co-grazing of carriers (sheep or wildebeest) and susceptible animals (cattle or buffaloes) is one of the MCF control actions that has been considered [31, 48]. Genotypes obtained from SA strains showed random distribution in the different provinces of SA. Since the number of samples analyzed for most of the genes under investigation was limited and varied for different provinces, no conclusive findings could be drawn in this regard. However, a reasonable number of samples were analyzed for the Ov 8 ex2 gene and the findings suggest that the distribution of Ov 8 ex2 genotypes is likely to be random among the various provinces. Ov 8 ex2 sequences analysis also showed that the distribution of genotype 2 identified in SA samples was independent of the period of collection as it was observed in samples collected in 2007, 2008 as well as in 2009. Nonetheless, a higher number of samples from each province will have to be analyzed in order to determine the actual distribution pattern of genotypes of each gene.

B cell epitopes are important in vaccine development because they are recognized by membrane bound antibodies present on the outer surface of B lymphocytes and are regarded as antigenic determinants [52]. Furthermore, these epitopes can also react with antibodies in serodiagnostic tests [30, 37, 53]. The predicted B-cell epitopes identified for Ov 7, Ov 8 ex2 and ORF 27 genes seemed conserved which is an advantage for serodiagnostic test development and vaccine design.

Proteins successfully used for improving sero-diagnostic tests and subunit vaccines have properties such as signal peptide, situated in the outer membrane, may be involved in metabolic process and ATP biosynthesis [54]. In addition, proteins responsible for transport and proteolysis have also been shown to have the ability to react with antibodies [54, 55]. The glycoproteins encoded by Ov 7 and Ov 8 ex2 genes of OvHV-2 were predicted to be responsible for attachment of the virus to host receptor cells [17] indicating that they are surface glycoproteins, and the protein encoded by ORF 27 were predicted to be responsible for transmission of the virus from one cell to another [17] which is a transport activity. The Ov 7, Ov 8 ex2 and ORF 27 gene products can be good candidates for serodiagnosis and subunit vaccine candidates because their encoded glycoproteins possess the features required for good candidates for serodiagnosis or subunit vaccine development including the presence of transmembrane domain, situated in the outer membrane surface, and possession of signal peptides.

This is the first report on characterization and genotyping of OvHV-2 strains circulating in SA. The information obtained has given an insight on the level of genetic diversity among these strains which can be used to improve the serodiagnosis of the virus by developing more specific serodiagnostic tools that will take into consideration the genetic diversity that exists between OvHV-2 strains. Moreover, genotypes based on ORF 27 and ORF 73 genes were able to differentiate OvHV-2 strains based on the geographical origin, which can be useful for future epidemiological studies. In the interest of controlling SA-MCF in SA, the information can assist veterinary authorities to follow the distribution patterns of specific genotypes of OvHV-2 strains, particularly those linked to virulence, and establish factors responsible for the spread of the virus from one region to another, such as animal trade or transportation across provinces for socio-economic reasons. This could lead to the government re-considering the introduction of the former legislation of the Animal Diseases Act, 1984 used for control of MCF in SA [31].

## Conclusions

The Ov 8 ex2, Ov 7, ORF 27 and ORF 73 genes could be amplified with the primers designed and PCR assays developed. These PCR assays have a potential for use in the detection of OvHV-2 infection, but, require further optimization and validation to improve the assay sensitivity.

The findings have provided preliminary information on the diversity of OvHV-2 strains based on Ov 8 ex2, Ov 7, ORF 27 and ORF 73 genes. The nucleotide and derived amino acid sequences of the selected OvHV-2 genes from strains from SA can serve as references for future studies on aspects such as epidemiology and bioinformatics. Furthermore, findings of this study can be used for the selection of gene candidates for the development of diagnostic assays and vaccines for the prevention of OvHV-2 infection. It seems unlikely that variations on the protein sequences of the investigated genes would affect the function of the gene products or antibody binding activities of each protein because of the predicted conserved B-cell epitopes observed on the amino acid sequences of the glycoproteins coded by the selected genes.

## Supporting Information

S1 Table Average sequence identities determined for the Ov 7 nucleotide and amino sequences obtained between South African Ov 7 sequences compared to reference sequences. (PDF)

S2 Table Average sequence identities determined for the Ov 8 ex2 nucleotide (Figure A) and derived amino acid (Figure B) sequences obtained between South African OvHV-2 strains compared to reference strains. (PDF)

S3 Table Average sequence identities determined for the ORF 27 nucleotide and derived amino acid sequences obtained between South African OvHV-2 strains compared to reference strains. (PDF)

S4 Table Average sequence identities for the ORF 73 nucleotide and derived amino acid sequences obtained between South African OvHV-2 strains compared to reference strains. (PDF)
