# Supplementary material for: Molecular Analysis of South African Ovine Herpesvirus 2 Strains Based on Selected Glycoprotein and Tegument Genes
Source: PLoS One. 2016 Mar 22;11(3):e0147019. doi: 10.1371/journal.pone.0147019 (PMC4803344; doi:10.1371/journal.pone.0147019)
Supplement: S1 Table — (PDF) [file pone.0147019.s001.pdf]

S1 Table. Average sequence identities determined for the Ov 7 nucleotide and amino sequences obtained between South African Ov 7 sequences compared to reference sequences.

	Nucleotide							Derived amino acid						
	**1**	**2**	**3**	**4**	**5**	**6**	**7**	**1**	**2**	**3**	**4**	**5**	**6**	**7**
Ov7/AY839756/Ref (1)	ID	1.000	1.000	0.988	0.988	0.997	0.997	ID	1.000	1.000	0.966	0.966	0.991	0.991
Ov7/NC007646/Ref (2)	1.000	ID	1.000	0.988	0.988	0.997	0.997	1.000	ID	1.000	0.966	0.966	0.991	0.991
Ov7/DQ198083/Ref (3)	1.000	1.000	ID	0.988	0.988	0.997	0.997	1.000	1.000	ID	0.966	0.966	0.991	0.991
Ov7-22/WC/2009 (4)	0.988	0.988	0.988	ID	1.000	0.986	0.986	0.966	0.966	0.966	1.000	ID	0.958	0.958
Ov7-11/WC/2008 (5)	0.988	0.988	0.988	1.000	ID	0.986	0.986	0.966	0.966	0.966	ID	1.000	0.958	0.958
Ov7-28/EC/2007 (6)	0.997	0.997	0.997	0.986	0.986	ID	1.000	0.991	0.991	0.991	0.958	0.958	ID	1.000
Ov7-10/MP/2007 (7)	0.997	0.997	0.997	0.986	0.986	1.000	ID	0.991	0.991	0.991	0.958	0.958	1.000	ID

The shaded cells contain values comparing SA sequences to reference sequences.
