# Supplementary material for: Molecular Analysis of South African Ovine Herpesvirus 2 Strains Based on Selected Glycoprotein and Tegument Genes
Source: PLoS One. 2016 Mar 22;11(3):e0147019. doi: 10.1371/journal.pone.0147019 (PMC4803344; doi:10.1371/journal.pone.0147019)
Supplement: S2 Table — (PDF) [file pone.0147019.s002.pdf]

S2 Table. Average sequence identities determined for the Ov 8 ex2 nucleotide (A) and derived amino acid (B) sequences obtained between South African OvHV-2 strains compared to reference strains.

A

Nucleotide sequence ID	1	2	3	4	5	6	7	8	9	10	11	12	13	14	15	16	17	18	19	20	21	22	23	24	25
Ov8 ex2/NC007646/Ref (1)	ID	1.000	0.995	0.985	0.985	0.980	0.980	0.975	0.960	0.960	0.955	0.960	0.965	0.960	0.960	0.945	0.960	0.955	0.960	0.960	0.960	0.960	0.955	0.955	0.925
Ov8 ex2/AY839756/Ref (2)	1.000	ID	0.995	0.985	0.985	0.980	0.980	0.975	0.960	0.960	0.955	0.960	0.965	0.960	0.960	0.945	0.960	0.955	0.960	0.960	0.960	0.960	0.955	0.955	0.925
Ov8 ex2/DQ198083/Ref (3)	0.995	0.995	ID	0.990	0.990	0.985	0.985	0.980	0.965	0.965	0.960	0.965	0.970	0.965	0.965	0.950	0.965	0.960	0.965	0.965	0.965	0.965	0.960	0.960	0.930
Ov8 ex2-24/WC/2008 (4)	0.985	0.985	0.990	ID	0.980	0.975	0.975	0.970	0.955	0.955	0.950	0.955	0.960	0.955	0.955	0.940	0.955	0.960	0.955	0.955	0.955	0.955	0.950	0.950	0.920
Ov8 ex2-11/WC/2008 (5)	0.985	0.985	0.990	0.980	ID	0.985	0.995	0.990	0.965	0.960	0.955	0.960	0.965	0.960	0.960	0.945	0.960	0.955	0.960	0.960	0.960	0.960	0.960	0.960	0.930
Ov8 ex2-8/EC/2007 (6)	0.980	0.980	0.985	0.975	0.985	ID	0.980	0.975	0.960	0.955	0.950	0.955	0.960	0.955	0.955	0.950	0.955	0.950	0.955	0.955	0.955	0.955	0.955	0.955	0.925
Ov8 ex2-12/G/2008 (7)	0.980	0.980	0.985	0.975	0.995	0.980	ID	0.995	0.970	0.955	0.950	0.955	0.960	0.955	0.955	0.940	0.955	0.950	0.955	0.955	0.955	0.955	0.955	0.955	0.925
Ov8 ex2-1/FS/2009 (8)	0.975	0.975	0.980	0.970	0.990	0.975	0.995	ID	0.965	0.960	0.945	0.950	0.955	0.950	0.950	0.935	0.950	0.945	0.950	0.950	0.950	0.950	0.960	0.960	0.930
Ov8 ex2-13/WC/2009 (9)	0.960	0.960	0.965	0.955	0.965	0.960	0.970	0.965	ID	0.975	0.970	0.975	0.980	0.975	0.975	0.960	0.975	0.970	0.975	0.975	0.975	0.975	0.985	0.985	0.955
Ov8 ex2-3/FS/2008 (10)	0.960	0.960	0.965	0.955	0.960	0.955	0.955	0.960	0.975	ID	0.985	0.975	0.980	0.975	0.975	0.960	0.975	0.970	0.975	0.975	0.975	0.975	0.980	0.980	0.950
Ov8 ex2-16/WC/2008 (11)	0.955	0.955	0.960	0.950	0.955	0.950	0.950	0.945	0.970	0.985	ID	0.970	0.975	0.970	0.970	0.955	0.970	0.965	0.970	0.970	0.970	0.970	0.965	0.965	0.935
Ov8 ex2-37/WC/2009 (12)	0.960	0.960	0.965	0.955	0.960	0.955	0.955	0.950	0.975	0.975	0.970	ID	0.994	0.989	0.989	0.974	0.989	0.984	0.989	0.989	0.989	0.989	0.970	0.970	0.940
Ov8 ex2-38/WC/2009 (13)	0.965	0.965	0.970	0.960	0.965	0.960	0.960	0.955	0.980	0.980	0.975	0.994	ID	0.994	0.994	0.979	0.994	0.989	0.994	0.994	0.994	0.994	0.975	0.975	0.945
Ov8 ex2-27/EC/2007 (14)	0.960	0.960	0.965	0.955	0.960	0.955	0.955	0.950	0.975	0.975	0.970	0.989	0.994	ID	1.000	0.984	1.000	0.994	1.000	1.000	1.000	1.000	0.980	0.980	0.950
Ov8 ex2-28/EC/2007 (15)	0.960	0.960	0.965	0.955	0.960	0.955	0.955	0.950	0.975	0.975	0.970	0.989	0.994	1.000	ID	0.984	1.000	0.994	1.000	1.000	1.000	1.000	0.980	0.980	0.950
Ov8 ex2-2/FS/2009 (16)	0.945	0.945	0.950	0.940	0.945	0.950	0.940	0.935	0.960	0.960	0.955	0.974	0.979	0.984	0.984	ID	0.984	0.979	0.984	0.984	0.984	0.984	0.965	0.965	0.935
Ov8 ex2-5/NW/2008 (17)	0.960	0.960	0.965	0.955	0.960	0.955	0.955	0.950	0.975	0.975	0.970	0.989	0.994	1.000	1.000	0.984	ID	0.994	1.000	1.000	1.000	1.000	0.980	0.980	0.950
Ov8 ex2-15/WC/2009 (18)	0.955	0.955	0.960	0.960	0.955	0.950	0.950	0.945	0.970	0.970	0.965	0.984	0.989	0.994	0.994	0.979	0.994	ID	0.994	0.994	0.994	0.994	0.975	0.975	0.945
Ov8 ex2-40/G/2009 (19)	0.960	0.960	0.965	0.955	0.960	0.955	0.955	0.950	0.975	0.975	0.970	0.989	0.994	1.000	1.000	0.984	1.000	0.994	ID	1.000	1.000	1.000	0.980	0.980	0.950
Ov8 ex2-34/NC/2009 (20)	0.960	0.960	0.965	0.955	0.960	0.955	0.955	0.950	0.975	0.975	0.970	0.989	0.994	1.000	1.000	0.984	1.000	0.994	1.000	ID	1.000	1.000	0.980	0.980	0.950
Ov8 ex2-35/NC/2009 (21)	0.960	0.960	0.965	0.955	0.960	0.955	0.955	0.950	0.975	0.975	0.970	0.989	0.994	1.000	1.000	0.984	1.000	0.994	1.000	1.000	ID	1.000	0.980	0.980	0.950
Ov8 ex2-42/FS/2009 (22)	0.960	0.960	0.965	0.955	0.960	0.955	0.955	0.950	0.975	0.975	0.970	0.989	0.994	1.000	1.000	0.984	1.000	0.994	1.000	1.000	1.000	ID	0.980	0.980	0.950
Ov8 ex2-33/KZN/2009 (23)	0.955	0.955	0.960	0.950	0.960	0.955	0.955	0.960	0.985	0.980	0.965	0.970	0.975	0.980	0.980	0.965	0.980	0.975	0.980	0.980	0.980	0.980	ID	1.000	0.970
Ov8 ex2-39/KZN/2009 (24)	0.955	0.955	0.960	0.950	0.960	0.955	0.955	0.960	0.985	0.980	0.965	0.970	0.975	0.980	0.980	0.965	0.980	0.975	0.980	0.980	0.980	0.980	1.000	ID	0.970
Ov8 ex2-36/NW/2009 (25)	0.925	0.925	0.930	0.920	0.930	0.925	0.925	0.930	0.955	0.950	0.935	0.940	0.945	0.950	0.950	0.935	0.950	0.945	0.950	0.950	0.950	0.950	0.970	0.970	ID

B

Amino acid sequence ID	1	2	3	4	5	6	7	8	9	10	11	12	13	14	15	16	17	18	19	20	21	22	23	24	25
Ov8 ex2/NC007646/Ref (1)	ID	1.000	0.985	0.970	0.970	0.970	0.970	0.970	0.910	0.955	0.955	0.970	0.970	0.970	0.970	0.940	0.970	0.970	0.970	0.970	0.970	0.970	0.910	0.910	0.910
Ov8 ex2/AY839756/Ref (2)	1.000	ID	0.985	0.970	0.970	0.970	0.970	0.970	0.910	0.955	0.955	0.970	0.970	0.970	0.970	0.940	0.970	0.970	0.970	0.970	0.970	0.970	0.910	0.910	0.910
Ov8 ex2/DQ198083/Ref (3)	0.985	0.985	ID	0.985	0.985	0.985	0.985	0.985	0.925	0.970	0.970	0.985	0.985	0.985	0.985	0.955	0.985	0.985	0.985	0.985	0.985	0.985	0.925	0.925	0.925
Ov8 ex2-24/WC/2008 (4)	0.970	0.970	0.985	ID	0.970	0.970	0.970	0.970	0.910	0.955	0.955	0.970	0.970	0.970	0.970	0.940	0.970	0.970	0.970	0.970	0.970	0.970	0.910	0.910	0.910
Ov8 ex2-11/WC/2008 (5)	0.970	0.970	0.985	0.970	ID	0.970	1.000	1.000	0.910	0.955	0.955	0.970	0.970	0.970	0.970	0.940	0.970	0.970	0.970	0.970	0.970	0.970	0.910	0.910	0.910
Ov8 ex2-8/EC/2007 (6)	0.970	0.970	0.985	0.970	0.970	ID	0.970	0.970	0.910	0.955	0.955	0.970	0.970	0.970	0.970	0.955	0.970	0.970	0.970	0.970	0.970	0.970	0.910	0.910	0.910
Ov8 ex2-12/G/2008 (7)	0.970	0.970	0.985	0.970	1.000	0.970	ID	1.000	0.910	0.955	0.955	0.970	0.970	0.970	0.970	0.940	0.970	0.970	0.970	0.970	0.970	0.970	0.910	0.910	0.910
Ov8 ex2-1/FS/2009 (8)	0.970	0.970	0.985	0.970	1.000	0.970	1.000	ID	0.910	0.955	0.955	0.970	0.970	0.970	0.970	0.940	0.970	0.970	0.970	0.970	0.970	0.970	0.910	0.910	0.910
Ov8 ex2-13/WC/2009 (9)	0.910	0.910	0.925	0.910	0.910	0.910	0.910	0.910	ID	0.911	0.911	0.925	0.925	0.925	0.925	0.895	0.925	0.925	0.925	0.925	0.925	0.925	1.000	1.000	1.000
Ov8 ex2-3/FS/2008 (10)	0.955	0.955	0.970	0.955	0.955	0.955	0.955	0.955	0.911	ID	1.000	0.985	0.985	0.985	0.985	0.955	0.985	0.985	0.985	0.985	0.985	0.985	0.911	0.911	0.911
Ov8 ex2-16/WC/2008 (11)	0.955	0.955	0.970	0.955	0.955	0.955	0.955	0.955	0.911	1.000	ID	0.985	0.985	0.985	0.985	0.955	0.985	0.985	0.985	0.985	0.985	0.985	0.911	0.911	0.911
Ov8 ex2-37/WC/2009 (12)	0.970	0.970	0.985	0.970	0.970	0.970	0.970	0.970	0.925	0.985	0.985	ID	1.000	1.000	1.000	0.969	1.000	1.000	1.000	1.000	1.000	1.000	0.925	0.925	0.925
Ov8 ex2-38/WC/2009 (13)	0.970	0.970	0.985	0.970	0.970	0.970	0.970	0.970	0.925	0.985	0.985	1.000	ID	1.000	1.000	0.969	1.000	1.000	1.000	1.000	1.000	1.000	0.925	0.925	0.925
Ov8 ex2-27/EC/2007 (14)	0.970	0.970	0.985	0.970	0.970	0.970	0.970	0.970	0.925	0.985	0.985	1.000	1.000	ID	1.000	0.969	1.000	1.000	1.000	1.000	1.000	1.000	0.925	0.925	0.925
Ov8 ex2-28/EC/2007 (15)	0.970	0.970	0.985	0.970	0.970	0.970	0.970	0.970	0.925	0.985	0.985	1.000	1.000	1.000	ID	0.969	1.000	1.000	1.000	1.000	1.000	1.000	0.925	0.925	0.925
Ov8 ex2-2/FS/2009 (16)	0.940	0.940	0.955	0.940	0.940	0.955	0.940	0.940	0.895	0.955	0.955	0.969	0.969	0.969	0.969	ID	0.969	0.969	0.969	0.969	0.969	0.969	0.895	0.895	0.895
Ov8 ex2-5/NW/2008 (17)	0.970	0.970	0.985	0.970	0.970	0.970	0.970	0.970	0.925	0.985	0.985	1.000	1.000	1.000	1.000	0.969	ID	1.000	1.000	1.000	1.000	1.000	0.925	0.925	0.925
Ov8 ex2-15/WC/2009 (18)	0.970	0.970	0.985	0.970	0.970	0.970	0.970	0.970	0.925	0.985	0.985	1.000	1.000	1.000	1.000	0.969	1.000	ID	1.000	1.000	1.000	1.000	0.925	0.925	0.925
Ov8 ex2-40/G/2009 (19)	0.970	0.970	0.985	0.970	0.970	0.970	0.970	0.970	0.925	0.985	0.985	1.000	1.000	1.000	1.000	0.969	1.000	1.000	ID	1.000	1.000	1.000	0.925	0.925	0.925
Ov8 ex2-34/NC/2009 (20)	0.970	0.970	0.985	0.970	0.970	0.970	0.970	0.970	0.925	0.985	0.985	1.000	1.000	1.000	1.000	0.969	1.000	1.000	1.000	ID	1.000	1.000	0.925	0.925	0.925
Ov8 ex2-35/NC/2009 (21)	0.970	0.970	0.985	0.970	0.970	0.970	0.970	0.970	0.925	0.985	0.985	1.000	1.000	1.000	1.000	0.969	1.000	1.000	1.000	1.000	ID	1.000	0.925	0.925	0.925
Ov8 ex2-42/FS/2009 (22)	0.970	0.970	0.985	0.970	0.970	0.970	0.970	0.970	0.925	0.985	0.985	1.000	1.000	1.000	1.000	0.969	1.000	1.000	1.000	1.000	1.000	ID	0.925	0.925	0.925
Ov8 ex2-33/KZN/2009 (23)	0.910	0.910	0.925	0.910	0.910	0.910	0.910	0.910	1.000	0.911	0.911	0.925	0.925	0.925	0.925	0.895	0.925	0.925	0.925	0.925	0.925	0.925	ID	1.000	1.000
Ov8 ex2-39/KZN/2009 (24)	0.910	0.910	0.925	0.910	0.910	0.910	0.910	0.910	1.000	0.911	0.911	0.925	0.925	0.925	0.925	0.895	0.925	0.925	0.925	0.925	0.925	0.925	1.000	ID	1.000
Ov8 ex2-36/NW/2009 (25)	0.910	0.910	0.925	0.910	0.910	0.910	0.910	0.910	1.000	0.911	0.911	0.925	0.925	0.925	0.925	0.895	0.925	0.925	0.925	0.925	0.925	0.925	1.000	1.000	ID

The shaded cells contain values comparing SA sequences to reference sequences.
