# Supplementary material for: Molecular Analysis of South African Ovine Herpesvirus 2 Strains Based on Selected Glycoprotein and Tegument Genes
Source: PLoS One. 2016 Mar 22;11(3):e0147019. doi: 10.1371/journal.pone.0147019 (PMC4803344; doi:10.1371/journal.pone.0147019)
Supplement: S3 Table — (PDF) [file pone.0147019.s003.pdf]

S3 Table. Average sequence identities determined for the ORF 27 nucleotide and derived amino acid sequences obtained between South African OvHV-2 strains compared to reference strains.

	Nucleotide								Derived amino acid							
	**1**	**2**	**3**	**4**	**5**	**6**	**7**	**8**	**1**	**2**	**3**	**4**	**5**	**6**	**7**	**8**
ORF27/NC007646/Ref (1)	ID	1.000	0.986	0.976	0.976	0.976	0.976	0.976	ID	1.000	0.979	0.897	0.897	0.897	0.897	0.897
ORF27/AY839756/Ref (2)	1.000	ID	0.986	0.976	0.976	0.976	0.976	0.976	1.000	ID	0.979	0.897	0.897	0.897	0.897	0.897
ORF27/DQ198083/Ref (3)	0.986	0.986	ID	0.963	0.963	0.963	0.963	0.963	0.979	0.979	ID	0.877	0.877	0.877	0.877	0.877
ORF27-6/FS/2009 (4)	0.976	0.976	0.963	ID	1.000	1.000	1.000	1.000	0.897	0.897	0.877	ID	1.000	1.000	1.000	1.000
ORF27-3/FS/2008 (5)	0.976	0.976	0.963	1.000	ID	1.000	1.000	1.000	0.897	0.897	0.877	1.000	ID	1.000	1.000	1.000
ORF27-37/WC/2009 (6)	0.976	0.976	0.963	1.000	1.000	ID	1.000	1.000	0.897	0.897	0.877	1.000	1.000	ID	1.000	1.000
ORF27-1/FS/2009 (7)	0.976	0.976	0.963	1.000	1.000	1.000	ID	1.000	0.897	0.897	0.877	1.000	1.000	1.000	ID	1.000
ORF27-13/WC/2009 (8)	0.976	0.976	0.963	1.000	1.000	1.000	1.000	ID	0.897	0.897	0.877	1.000	1.000	1.000	1.000	ID

The shaded cells contain values comparing SA sequences to reference sequences.
