# Supplementary material for: Molecular Analysis of South African Ovine Herpesvirus 2 Strains Based on Selected Glycoprotein and Tegument Genes
Source: PLoS One. 2016 Mar 22;11(3):e0147019. doi: 10.1371/journal.pone.0147019 (PMC4803344; doi:10.1371/journal.pone.0147019)
Supplement: S4 Table — (PDF) [file pone.0147019.s004.pdf]

S4 Table. Average sequence identities for the ORF 73 nucleotide and derived amino acid sequences obtained between South African OvHV-2 strains compared to reference strains.

	Nucleotide						Derived amino acid					
	**1**	**2**	**3**	**4**	**5**	**6**	**1**	**2**	**3**	**4**	**5**	**6**
ORF73/AY839756 (1)	ID	1.000	0.854	0.837	0.836	0.836	ID	1.000	0.851	0.832	0.830	0.830
ORF73/NC007646 (2)	1.000	ID	0.854	0.837	0.836	0.836	1.000	ID	0.851	0.832	0.830	0.830
ORF73/DQ198083 (3)	0.854	0.854	ID	0.971	0.970	0.970	0.851	0.851	ID	0.966	0.964	0.964
ORF73-36/NW/2009 (4)	0.837	0.837	0.971	ID	0.998	0.998	0.832	0.832	0.966	ID	0.996	0.996
ORF73-10/MP/2007 (5)	0.836	0.836	0.970	0.998	ID	1.000	0.830	0.830	0.964	0.996	ID	1.000
ORF73-3/FS/2008 (6)	0.836	0.836	0.970	0.998	1.000	ID	0.830	0.830	0.964	0.996	1.000	ID

The shaded cells contain values comparing SA sequences to reference sequences.
